# Challenges in assessing biological recovery from acidification in Swedish lakes

**DOI:** 10.1007/s13280-014-0559-y

**Published:** 2014-11-15

**Authors:** Kerstin Holmgren

**Affiliations:** Department of Aquatic Resources, Institute of Freshwater Research, Swedish University of Agricultural Sciences, Stångholmsvägen 2, 178 93 Drottningholm, Sweden

**Keywords:** Acidification, Lakes, Time series, Biological recovery, Dominant species

## Abstract

Since the 1980s, Swedish lakes have in general become less acidified. Assessment of biological recovery is, however, hampered by poor pre-acidification data, confounding effects of climate change, and few lakes with annual sampling of fish and other organisms. Only three critically acidified, but non-limed, lakes had two decades of fish monitoring. The lakes had not yet recovered to pre-industrial chemical targets. Fish had low species richness compared to other organism groups. Roach (*Rutilus rutilus*) and/or European perch (*Perca fluviatilis*) were the dominant fish species, and the acid-sensitive roach had been lost from one of the lakes. Calcium decreased, possibly approaching pre-acidification concentrations, but exceeded minimum levels needed to sustain some *Daphnia* species. High or increasing levels of total organic carbon, likely due to reduced acidification and climate change, might influence the biological communities in unexpected ways, for example, facilitating more frequent occurrence of the invasive algae *Gonyostomum semen*.

**Electronic supplementary material:**

The online version of this article (doi:10.1007/s13280-014-0559-y) contains supplementary material, which is available to authorized users.

## Introduction

During the twentieth century, anthropogenic acidification was a main reason for both direct and indirect changes in ecosystems of lakes and streams in the northern hemisphere (Almer 1974; Dillon et al. 1984; Schindler 1988). Recruitment decreased in acid-sensitive species, and some populations were completely lost from lakes and river reaches. At the community level, species richness decreased, competitive and predatory interactions were modified, and reduced decomposition rates were sometimes observed (Appelberg et al. 1993; Stenson et al. 1993).

International conventions in the 1980s led to massive reductions in SO_2_ emission, followed by decreased sulfur deposition, and chemical recovery was consistently observed in North American and European lakes and streams (Stoddard et al. 1999). Sulfate concentrations decreased in the majority of lakes in Sweden and other Nordic countries, and acid-neutralizing capacity increased (Skjelkvåle et al. 2001; Fölster et al. 2005; Garmo et al. 2014).

Scattered examples of biological recovery have been reported from Canadian and Norwegian lakes (Gunn and Sandøy 2003; Hesthagen et al. 2011). Loss of acid-sensitive species is one of the expected effects of acidification, often indirectly inferred by observation of lower species richness in acidic compared to circum-neutral lakes (e.g., Almer et al. 1974; Persson 2008). Increased species richness within taxonomic groups is therefore an expected, and sometimes observed, response to chemical recovery (e.g., Keller and Yan 1991; Findlay 2003). Observed species richness, however, depends on spatial and temporal scales of sampling, as well as on dispersal and colonization rates of targeted organisms. For example, small and passively dispersing organisms tend to occur everywhere with high local species richness, in contrast to low local richness of larger and actively dispersing fish (Fenchel and Finlay 2004).

There is an increasing awareness that aquatic organisms may face multiple or unexpected stressors in lakes recovering from acidification (Palmer and Yan 2013). Phytoplankton and benthic invertebrates responded in complex ways to decreasing acidity and inter-annual variability in climate factors in Swedish lakes (Stendera and Johnson 2008; Johnson and Angeler 2010; Angeler and Johnson 2012).

Changes in mobile anions are important for the Ca^2+^ leakage from soils to surface waters (Reuss and Johnson 1986), e.g., increasing Ca concentration and ionic strength in surface waters during acidification. Ca is therefore expected to decrease along with decreasing SO_4_ as surface waters recover from acidification. Decreasing Ca might, however, also pose a threat to calcium-rich species (Jeziorski et al. 2008), like *Daphnia* spp. (Wærvågen et al. 2002).

Dissolved organic carbon (DOC) is increasing in many North American and northern European lakes, partly as a consequence of decreasing sulfate and calcium levels (Monteith et al. 2007), but also affected by increasing temperature and/or changed hydrological regimes. Reduction in H^+^ means more negatively charged organic carbon, leading to increased solubility of total organic carbon (TOC), indicating a close coexistence of natural and anthropogenic acidification (Erlandsson et al. 2011). Increasing DOC has generally retarded recovery defined as pH increase (Erlandsson et al. 2010), and high TOC content characterizes non-recovering lakes when compared to recovering lakes (Vuorenmaa and Forsius 2008). High DOC may affect organisms in different ways, e.g., negatively influence light conditions for primary producers (Karlsson et al. 2009) and visually hunting fish (Rask et al. 2014), or positively subsidizing heterotrophic bacteria and zooplankton in the pelagic zone (Jones et al. 2012).

Biological recovery should ideally be assessed in relation to a specified pre-acidification condition, for example defined as abundance, recruitment and/or growth of locally dominating species. A decision tree has been suggested by Yan et al. (2003) to identify different bottlenecks for recovery of selected species (i.e., inadequate water quality, no available colonists, too few colonists to reach a viable population size, community-level confounding factors). For lost populations (as fish species), active reintroduction is needed to reestablish pre-acidified food web structures (Gunn and Mills 1998).

To mitigate acidification, until reduced atmospheric deposition could facilitate natural recovery, a large-scale Swedish liming program started in 1982. This program expanded to include up to 7500 lakes with repeated limestone treatment (Svenson et al. 1995). The liming targeted pH >6 and alkalinity >0.1 meq L^−1^. Along with the liming, a large-scale monitoring program was launched in non-limed and soft water boreal lakes, with initial pH both below and above 6. Regular monitoring of biological communities began in 1988 in 26 lakes (Persson 1996). Twelve of these lakes were later supplemented with annual fish sampling from 1994 (Holmgren 1999), and regular sampling of zooplankton started in different years in the same lakes.

Historical records of fish species occurrence indicated recent re-colonization of acid-sensitive roach (*Rutilus rutilus*) in a few chemically recovering Swedish lakes (Valinia et al. 2014). More commonly, assessment of biological recovery is hampered by no data at all representing pre-acidification communities, or just analysis of diatom shells in sediment profiles to infer historical pH (Guhren et al. 2003).

This study illustrates challenges in assessments of biological recovery within acidified Swedish lakes, by using available time series of fish and other organisms of acidified lakes in the national monitoring program. More specific objectives were to (1) examine species richness of fish species and other organism groups, (2) describe trends in populations of the dominant fish, as well as abiotic and biotic factors assumed to influence fish recruitment or growth, and (3) discuss potential bottlenecks for biological recovery.

## Materials and methods

### Study lakes

Twelve non-limed lakes in the national monitoring program had annual monitoring of fish communities. The lakes were distributed from latitude 56° to 68° and altitude between 56 and 488 m above sea level (Table 1). A considerable range in regional climate was indicated by annual mean air temperature from 7°C in lowland lakes in the south to −1°C at higher latitude in the north. The lakes were rather small, with lake area of 10–282 ha and maximum depth varying from 9 to 40 m.

**Table 1:** Some descriptors of 12 lakes in the national monitoring program. The lakes are sorted from south to north. Mean air temperature refers to regional annual mean during 1961–1990. Text in bold type indicates acidified lakes, based on historical and measured pH before annual fish sampling. Historical pH was reconstructed by Guhren et al. (2003) using two different calibration datasets (S = SWAP, N = Norrset). Measured pH (mean and minimum values) is based on 30–66 samples during 1983–1993

Lake name	Latitude	Altitude (m)	Lake area (ha)	Maximum depth (m)	Mean air temp. (°C)	Historical and measured pH		
						pH (S)	pH (N)	pH 1983–1993
**Brunnsjön**	**56.5973**	**98**	**10**	**13**	**7**	**5.9 (−)**	**6.1 (+++)**	**5.38 (4.84)**
Stora Skärsjön	56.6717	60	32	12	7			6.64 (6.12)
Fiolen	57.0922	226	156	10	6	6.4 (−)	6.8 (+++)	6.26 (5.82)
Allgjutten	57.9483	126	18	40	7	6.8 (+++)	7.1 (+++)	6.44 (5.63)
Fräcksjön	58.1485	56	28	15	7	6.6 (++)	6.9 (+++)	6.24 (5.56)
**Rotehogstjärnen**	**58.8155**	**121**	**16**	**9**	**6**	**5.7 (+++)**	**6.0 (+++)**	**5.21 (4.58)**
Stora Envättern	59.1154	62	37	11	6	6.5 (+++)	6.9 (+++)	6.43 (5.89)
**Övre Skärsjön**	**59.8377**	**219**	**169**	**32**	**5**	**6.4 (+++)**	**6.7 (+++)**	**5.36 (4.66)**
Stensjön	61.6435	268	59	9	4	6.0 (+++)	6.4 (+++)	6.20 (5.72)
Remmarsjön	63.8631	234	140	14	2	6.0 (++)	6.3 (+++)	6.24 (5.68)
Jutsajaure	67.0604	422	113	10	−1	6.1 (++)	6.7 (+++)	6.48 (6.03)
Abiskojaure	68.3074	488	282	35	−1	6.5 (+)	7.0 (+)	6.94 (6.64)

+++ = very good, ++ = good, + = acceptable, and −= bad analog

Historical pH was taken from Guhren et al. (2003), based on analyses of diatom shells in lake sediments (30 cm depth, approximate age 150–400 years). More recently, pH was measured during ten years before fish sampling. Only three of 12 lakes were identified as acidified study lakes, having mean pH <6 during 1983–1993, and recent pH less than historical pH (Table 1). The three acidified lakes were selected for further analyses.

### Data collection

Fish data from 1994 to 2013 were extracted from the National Register of Survey test-fishing (NORS), managed by the Department of Aquatic Resources. Fish were sampled in July or August, with multi-mesh Nordic gillnets in a depth-stratified design (CEN 2005). Depending on lake size, annual sampling effort was 8 or 40 benthic gillnet nights per lake. Data sets included number of fish and biomass (g) of each species caught in each gillnet, individual length (mm) of all fish, and also mass (g) and age for subsamples of dominating species (Holmgren 2013).

Physicochemical and biological data since 1994 were extracted from a national database managed by the Department of Aquatic Sciences and Assessment and analyzed by the accredited chemical and biological laboratories at the same department. Measurements of water quality, phytoplankton, and benthic invertebrates covered 19 years during 1994–2012 (Electronic Supplementary Material, Table S1). Zooplankton data from epilimnion (all lakes) and hypolimnion (the deepest lake) were available from 4 monthly samples per year during 13–19 years. Phytoplankton samples from 3 months (May, July and August) were selected for this study because they were consistently taken in all lakes and all 19 years of the study period. Benthic invertebrates were sampled once a year in autumn, most often one composite sample from each of three habitats (littoral, sublittoral and profundal), with all three habitats sampled in 16–17 years.

Water samples were taken almost monthly during the ice-free period, at one mid-station per lake at approximately 0.5 m depth. Samples were analyzed for pH, alkalinity, conductivity, Ca^2+^, SO_4_^2−^, total P, total N, water color (absorbance at 420 nm), total organic carbon (TOC), and aluminum (especially the inorganic form, Al_i_). All physicochemical analyses except analyses of Al_i_ were done at the accredited Geochemical laboratory at Department of Aquatic Sciences and Assessment, Swedish University of Agricultural Sciences. Al_i_ was analyzed at the Department of Applied Science (Stockholm University), nominally defined as the fraction of total monomeric Al retained by a cation exchange resin (Andrén and Rydin 2009). All analyses were done following international (ISO) or European (EN) standards when available (Wilander et al. 2003).

Zooplankton was collected with a Limnos sampler (4.3 L) at one mid-station per lake, sampling at 2-m depth intervals. The collected water was mixed in a bucket, separately for epilimnetic and hypolimnetic samples, and animals were collected on a 0.04 mm net (Persson 2008). For phytoplankton, a 2-m tube sampler was used in the epilimnion, at five sites per lake to get one composite sample (Stendera and Johnson 2008). Composite samples of benthic invertebrates included five 20 s replicates of kick sampling (littoral) or five Ekman grab samples (5 × 0.247 cm^2^), each in sublittoral and profundal sites. Animals were collected by a hand net or sieve with 0.5-mm mesh size (Stendera and Johnson 2008).

Organisms were recorded as species (fish) or the lowest possible taxonomic level, e.g., genus, family, or order, for some taxa of phytoplankton, zooplankton, and benthic invertebrates.

### Annual metrics of abiotic factors

Water temperature was averaged over 5 months (May–September) to represent the main growth season of dominant fish species. Other physicochemical variables were generally expressed as annual means of all sampled months (most often 8) and additionally as minimum pH and maximum Al_i_.

### Annual biological metrics

For each organism group, an annual species (or taxa) list was established and used to calculate species richness. Additionally, the annual species lists were combined to get cumulative number of observed species for each of the first to the final year of each time series.

Biomass of the dominating fish species was expressed as average biomass per benthic gillnet (Bpue, g/gillnet) in annual samples. Fish populations were also described by mean individual weight (Mean*W*, g) and proportion of young fish (% young, age 1+ to 3+) according to Holmgren (2013). Growth of perch (*Perca fluviatilis*) was expressed both as length at age 2+ (L2+ , mm) and as back-calculated length (bcL, mm) after the first year of life (Holmgren and Appelberg 2001). At age 1+ and 2+, perch was assumed to include more benthic invertebrates in its diet, compared to feeding primarily on zooplankton in the first year (e.g., Estlander et al. 2010).

Biomass of phytoplankton and zooplankton were expressed as annual averages of biovolume (mm^3^ m^−3^), to match one census per year for fish and benthic invertebrates. Zooplankton samples from epilimnion and hypolimnion were first pooled within months, and then annually averaged over four samples from April, May, July, and August. For phytoplankton, total biomass was retained along with % of total biomass for taxonomic classes exceeding 30 % of total biomass in some or all lakes and years. Biomass of zooplankton was separately calculated for Rotatoria, Cladocera, Calanoida, and Cyclopoida, and for the sum of all groups.

Biomass of littoral benthic invertebrates was unfortunately not available, but abundance was indicated by the number of individuals per standard kick sample. Biomass of benthic invertebrates in sublittoral and profundal habitats was expressed as g m^−2^. For littoral samples, abundance was also used separately for burrowing Chironomidae, for non-burrowing Crustacea, and collectively for the most abundant orders of large insect larvae (Ephemeroptera, Plecoptera, Trichoptera, Megaloptera, and Odonata). Biomass was also used for two dominant groups in profundal samples, i.e., Chironomidae and *Chaoborus flavicans* (the only observed species of Chaoboridae).

### Monotonic trends and correlations

Kendall’s correlation (*τ*) was used to test for monotonic temporal trends within lakes of selected abiotic and biological metrics, with *P* < 0.05 indicating a significant trend. Test results are given along with descriptive statistics in Electronic Supplementary Material, Table S1. Kendall’s correlation was also used to indicate significant correlations (*P* < 0.01) between biological and abiotic metrics. Pair-wise correlations were also tested between organisms at different trophic levels, again applying *P* < 0.01. In this explorative study, significance levels were set to detect potentially interesting relationships while being aware that many tests increase the risk for spurious relationships.

## Results

### Abiotic trends

**Fig. 1:**
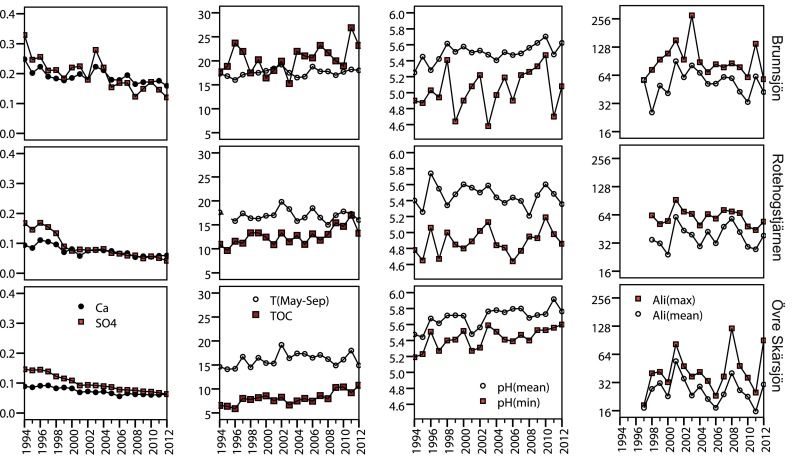
Time series of physical and chemical factors (annual mean or extreme values) in the three acidified lakes. Al_i_ is shown in log_2_ scale to enhance readability of relative differences between lakes and years. Units are meq L^−1^ for Ca and SO_4_, °C for T(May–Sep), mg L^−1^ for TOC, and μg L^−1^ for Al_i_

As expected, SO_4_ and Ca decreased significantly during 1994–2012 in each of the three acidified lakes (Fig. 1; Table S1, Electronic Supplementary Material). Lake Brunnsjön had the highest TOC level (mean 20.4 mg L^−1^) with no or weak increasing trend, while increasing TOC trends appeared in Lakes Rotehogstjärnen (10–17 mg L^−1^) and Övre Skärsjön (6–11 mg L^−1^). Mean pH increased in two lakes (Brunnsjön and Övre Skärsjön), but mean and minimum pH remained below 6 in all lakes and years. Total P tended to decrease in all lakes (ranges 8–18, 6–15 and 3–9 μg L^−1^). Water temperature fluctuated without significant trends. Among the selected abiotic metrics, only mean pH in Övre Skärsjön tended to correlate positively with temperature (*τ* = 0.406, *P* = 0.016). Mean and maximum Al_i_ did not change monotonically since measurements started in 1997. Al_i_ was consistently higher in Brunnsjön (mean 55 μg L^−1^) than in the other lakes (39 and 27 μg L^−1^).

### Species richness and biological trends

A total of only five fish species were observed in the acidified lakes, with three or five species observed at least once in each time series. Perch and Northern pike (*Esox lucius*) occurred in all lakes, but pike was not always found in the annual samples. Roach was caught every year in two lakes, and only once in the third lake. A few specimens of common bream (*Abramis abra*) and rudd (*Scardinus erythropthalmus*) were additionaly caught in most years in Lake Brunnsjön.

The low species richness of fish was in sharp contrast to cumulative numbers of taxa observed within lakes for the other organism groups (44–48 taxa of zooplankton, 97–123 taxa of phytoplankton, and 129–144 taxa of benthic invertebrates).

A total of 34 biological metrics were tested for trends, and 18 metrics changed significantly in at least one of the three lakes (detailed results in Electronic Supplementary Material, Table S1). Annual species richness did not change significantly for fish and phytoplankton, but increased for zooplankton and benthic invertebrates in two lakes.

**Fig. 2:**
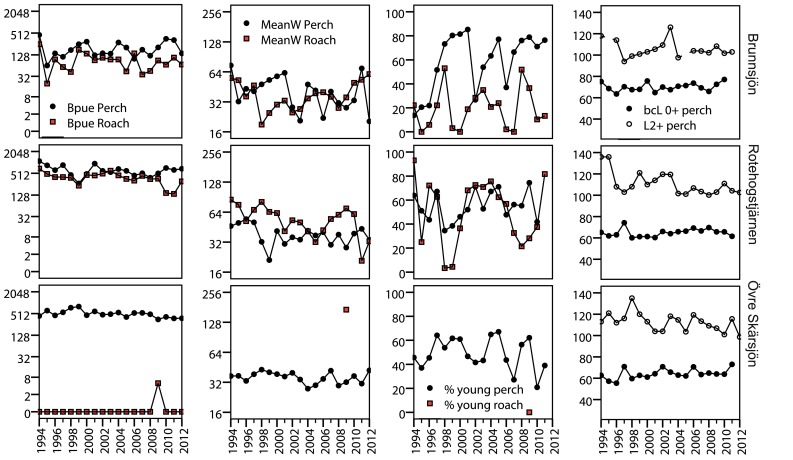
Time series of populations of perch and roach. Biomass (Bpue, g gillnet^−1^) and mean weight (Mean*W*, g) is shown in log_2_ scale to enhance readability of relative differences between fish species, lakes, and years. % young perch and roach = sum of % 1+ to 3+. bcL = back-calculated length at age 0+. L2+ = length at age 2+. The unit is mm for bcL 0+ and L2+

Perch BPUE decreased in the lake where only one individual roach (259 mm, 206 g, age 8+) was observed in the time series (Fig. 2). Roach BPUE and mean weight decreased in one of the lakes. Perch length at age 2+ decreased in Lake Övre Skärsjön. In contrast, growth of age 0+ increased in the same lake, indicating improved growth conditions in the presumed planktivorous stage.

**Fig. 3:**
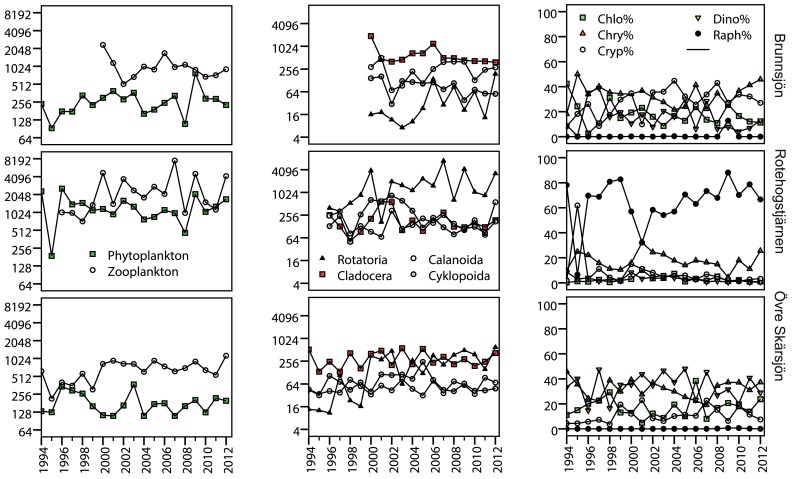
Time series of total biomass (mm^3^ m^−3^) of phytoplankton and zooplankton (*left*), biomass (mm^3^ m^−3^) within zooplankton groups (*middle*), and biomass proportions of dominant phytoplankton groups (*right*), i.e., Chlorophyceae (Chlo%), Chrysophyceae (Chrys%), Cryptophyceae (Cryp%), Dinophyceae (Dino%), and Raphidiophyceae (Raph%). Biomass is shown in log_2_ scale to enhance readability of relative differences between organism groups, lakes, and years

No monotonic trends were observed for total biomass of either phytoplankton or zooplankton (Fig. 3). The only increasing biomass trend of zooplankton was an increase of Rotatoria in the least humic lake, where it became a dominating group in the last years. The high biomass of Rotatoria in two of the three acidified lakes was heavily influenced by high biomass of the omnivorous species *Asplanchna priodonta*. Cladocera and cyclopoid copepods decreased in the most humic lake. The biomass proportion of Dinophyceae decreased in one lake, and Chrysophyceae and the mixotrophic *Gonyostomum semen* (order Raphidiophyceae) increased in another lake.

**Fig. 4:**
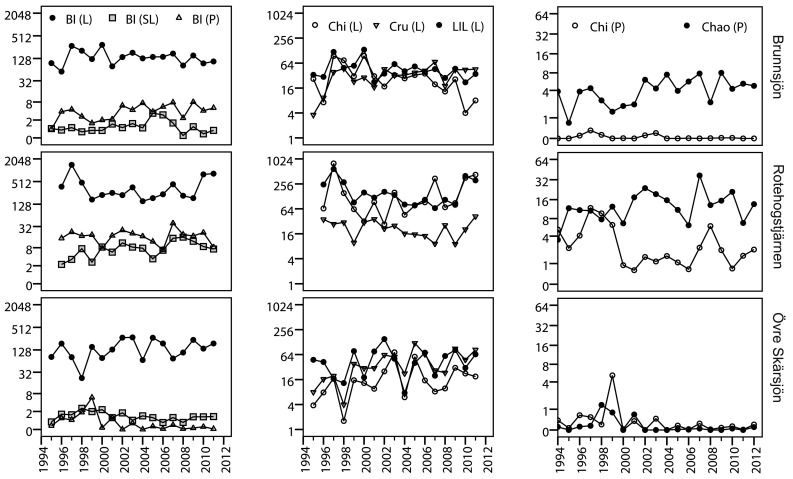
Time series of total biomass of benthic invertebrates (BI) in littoral (L, *n* sample^−1^), sublittoral (SL, g m^−2^), and profundal (P, g m^−2^) habitats, and biomass (g m^−2^) within dominating invertebrate groups, Chironomideae (Chi), Crustaceae (Cru), large insect larvae (LIL), and *Chaoborus flavicans* (Chao). Biomass is shown in log_2_ scale to enhance readability of relative differences between organism groups, lakes, and years

There were no temporal trends in the total abundance of littoral invertebrates (Fig. 4), but littoral Crustacea increased from low levels in Lakes Brunnsjön and Övre Skärsjön. Crustacea was almost exclusively *Asellus aquaticus*, with a second species *Gammarus pulex* observed only once in Lake Brunnsjön. Total biomass of sublittoral invertebrates increased in Lake Rotehogstjärnen. Biomass of profundal invertebrates increased in Lake Brunnsjön, but decreased in Lake Övre Skärsjön. *Chaoborus flavicans* was the dominant species in the profundal of Lakes Brunnsjön and Rotehogstjärnen, and its biomass increased in Lake Brunnsjön.

### Correlations with abiotic metrics

There were just a few direct relationships between biological and abiotic metrics (Kendall’s correlations, *P* < 0.01). The expected positive effect of water temperature (average May–Sep) on growth of perch 0+ was only significant in Lake Övre Skärsjön (*n* = 18, *τ* = 0.493, *P* = 0.004). There were no strong positive correlations between biologic metrics and Ca^2+^, but biomass of Rotatoria was negatively related to Ca^2+^ in Lake Övre Skärsjön (*n* = 19, *τ* = 0.532, *P* = 0.003). The only strong association between pH and biological metrics was a positive correlation between % young perch and mean pH in Lake Brunnsjön (*n* = 18, *τ* = 0.464, *P* = 0.007). Roach biomass in Lake Rotehogstjärnen was the only metric strongly and positively related to TOC (*n* = 19, *τ* = −0.509, *P* = 0.002).

### Comparisons between biological metrics

There were also just a few strong direct relationships between fish growth and biomass of potential prey or competitors, or between dominant groups of zooplankton or benthic invertebrates. Perch biomass in Lake Övre Skärsjön was positively related with growth of age 2+ perch (*n* = 19, *τ* = 0.512, *P* = 0.002). In the same lake, there were negative correlations between perch L2+ and biomass of Rotatoria (*n* = 19, *τ* = −0.476, *P* = 0.005), and between perch biomass and littoral Crustaceae (*n* = 17, *τ* = −0.480, *P* = 0.007). In Lake Rotehogstjärnen, first year growth of perch (bcL 0+) was positively correlated with calanoid copepods (*n* = 16, *τ* = 0.527, *P* = 0.005).

In littoral samples from Lake Övre Skärsjön, *A. aquaticus* was strongly correlated with Chironomideae (*n* = 17, *τ* = 0.701, *P* < 0.001). A similar covariance between Chironomidae and large insect larvae was observed in Brunnsjön (*n* = 17, *τ* = 0.603, *P* = 0.001) and Rotehogstjärnen (*n* = 16, *τ* = 0.510, *P* = 0.006). There was a positive correlation between Chironomideae and *C. flavicans* in the profundal habitat of Lake Övre Skärsjön (*n* = 19, *τ* = 0.499, *P* = 0.002).

## Discussion

### General abiotic trends

The three acidified study lakes followed expected patterns of decreasing sulfate and increasing pH along with decreasing Ca and increasing TOC, as also documented in other lakes in Europe and North America (e.g., Garmo et al. 2014). The studied lakes had, however, not yet recovered to pre-industrial chemical conditions. Moderate or high TOC levels probably retarded chemical recovery compared to other acidified, but less humic lakes (Vuorenmaa and Forsius 2008; Erlandsson et al. 2010).

### Species richness

Species richness increased from fish to zooplankton, and much higher richness in benthic invertebrates and phytoplankton. This is to expected, i.e., low local richness of large fish dispersing between connected waters, high local richness of small and passively dispersing plankton, and also high local richness of benthic invertebrates with flying dispersal stages (e.g., Fenchel and Finlay 2004). This also implies many possible predatory links from a few large predator species that might feed on preys of a wide spectrum of size (e.g., Woodward et al. 2005). As fish have low species richness, but potentially long generation times of a few dominating fish species, measurements of age and growth are recommended in standard monitoring (CEN 2005), for assessment of ecological status and trends according to the Water Framework Directive (European Union 2000).

### Trends and covariance with and between organism groups

This study indicated remarkably few, if any, direct effects of pH or Al_i_ on biological between-year variation, in spite of increasing pH in two of the three acidified lakes. There were also no clear biological effects of decreasing Ca^2+^. The negative correlation between biomass of Rotatoria and Ca^2+^ was not expected, but possibly an effect of unknown intercorrelated factors. Annual mean of Ca^2+^ generally exceeded 0.055 meq L^−1^ (or 1.1 mg L^−1^) throughout the study period, implying that low Ca^2+^ was not limiting the *Daphnia* species occurring in the present lakes (Wærvågen et al. 2002).

The three acidified lakes had high and/or increasing TOC concentrations. The increasing TOC is possibly a return to pre-acidification levels, after a historically low level during the last century (Monteith et al. 2007; Valinia et al. 2012). Zooplankton biomass exceeded phytoplankton biomass (Fig. 3), indicating high microbial carbon source for pelagic production (Jansson et al. 2000). In other humic and temperate lakes, terrestrial subsidies were needed to support animal production (Pace et al. 2004). Terrestrial insects may also be an important food source for pelagic fish (Mehner et al. 2005), but fish diet was not studied in the Swedish acidified lakes.

Increasing TOC was the most plausible explanation for decreasing growth of 0+ perch in a Finnish lake (Rask et al. 2014), explained by narrowing habitat with sufficient light for the visually feeding fish. Similarly, increasing TOC in Lakes Rotehogstjärnen and Övre Skärsjön might be one reason for decreasing length of age 2+ perch in the present study, e.g., by decreasing their foraging efficiency on benthic invertebrates. In contrast, growth of 0+ perch increased in limed or circum-neutral Swedish lakes (Jeppesen et al. 2012). In that study, first year growth was positively related to increasing water temperature, as observed here in Lake Övre Skärsjön, indicating that visual conditions were still good enough for perch in its zooplanktivourus stage.

A remarkable feature was that the large rotatorian *A. priodonta* dominated the zooplankton biomass in two of the three acidified lakes. *A. priodonta* is regularly occurring both in small humic lakes and in large eutrophic lakes, where it appears to be an opportunistic omnivore (Kappes et al. 2000; Oganjan et al. 2013). It is seldom identified as an important prey for fishes and may be underestimated in the diet analysis of fish larvae (Sutela and Huusko 2000).

Chironomids and *A. aquaticus* (Crustacea) are important prey for perch in humic lakes (Estlander et al. 2010), as here indicated by negative correlations between perch biomass and littoral Crustacea or Chironomidae in Lake Övre Skärsjön. Positive correlations between age 0+ perch growth and calanoid copepods, in two of the present lakes, indicate a common limiting factor rather than predator–prey interaction because planktivorous perch prefer cladocerans over copepods (e.g., Estlander et al. 2010). Causal interactions between fish and zooplankton might be hidden in the aggregated annual censuses used in this study, as well as when comparing mean values between lakes (Persson 2008).

*Gonyostomum semen* dominated phytoplankton biomass in one of the acidified lakes, and it occurred recently in low numbers in the two other lakes. It has been detected in an increasing number of Swedish lakes, sometimes causing algal blooms, and its biomass was inconsistently related to different environmental factors, e.g., including temperature, pH, or TOC (Angeler et al. 2012; Rengefors et al. 2012). The calanoid copepod *Eudiaptomus gracilis* and the cladoceran *Holopedium gibberum* can feed on *G. semen* at high rates (Johansson et al. 2013), but these species were usually not dominant zooplankton in the present lake dominated by *G. semen*.

### Potential bottlenecks for biological recovery

Insufficient water quality is an important bottleneck for biological recovery in the three Swedish acidified lakes, as recent annual mean pH was still below historical levels (Guhren et al. 2003). All three lakes had historical pH close to or well above 6, thereby indicating that acidity was not critically affecting roach recruitment (e.g., Degerman and Lingdell 1993; Rask et al. 1995).

For all organism groups except fish (Valinia et al. 2014), we currently do not know which species that dominated the pre-acidification communities. Currently increasing TOC levels might or might not reflect historical conditions (Valinia et al. 2012). Unfortunately, the increasing dominance of the invasive *G. semen* might be an unexpected and confounding factor. Faunal homogenization was already indicated in profundal invertebrates (Angeler and Johnson 2013), by increasing dominance of *C. flavicans* in lakes with *G. semen* blooms.

The presence of self-reproducing roach in Lakes Brunnsjön and Rotehogstjärnen, but not in Övre Skärsjön, is to some extent a paradox. Roach recruitment is, however, more irregular in Lakes Brunnsjön and Rotehogstjärnen than in circum-neutral lakes with comparable climate (Holmgren 2013). Surveys of many lakes revealed low probability of finding small roach in lakes with pH <6 (Holmgren and Buffam 2005), including Lakes Brunnsjön and Rotehogstjärnen as outstanding exceptions. Roach was present in Övre Skärsjön in 1949, but just a single specimen occurred in the present monitoring series. In 1994–1996, some old roach in Lake Rotehogstjärnen (maximum 26 years) indicated adult survival during many years with low recruitment. Strong year classes survived in 1991 and 1994, followed by annual recruitment 1999–2004. Roach eggs hatched successfully at pH above 5.5 in experiments, both in vivo and in vitro (Milbrink and Johansson 1975). Minimum pH was often below or close to this limit in Brunnsjön and Rotehogstjärnen, but more recently exceeded this level in Lake Övre Skärsjön.

The abiotic conditions in Lake Övre Skärsjön might now allow reintroduction of roach, as previously done in some limed lakes (Demandt and Björklund 2007), although occasionally high levels of Al_i_ might prevent successful reproduction every year. Presence of large piscivorous perch might further hinder re-establishment of roach, unless coexistence of perch and roach is mediated by the presence of pike as additional top predator (Persson et al. 2007). Pike was probably present throughout all years since pre-acidification time in the three acidified lakes. The current late summer sampling with gillnets (CEN 2005) was, however, not suitable to monitor any trends in pike abundance.

## Conclusion

Assessment of biological recovery from acidification in Swedish lakes is difficult as very few non-limed, acidified lakes have been immediately subjected to annual monitoring for fish and other organisms. Chemically, these lakes now follow generally observed trends in response to decreasing acidification and climate change. Biomass or growth of some dominating species responded as expected to increasing TOC levels and not to decreasing acidity. The abiotic conditions might now allow establishment of a previously lost roach population by re-introducing adult roach to this lake.

## Electronic supplementary material

Below is the link to the electronic supplementary material.

Supplementary material 1 (PDF 72 kb)
