# Supplementary material for: Challenges in assessing biological recovery from acidification in Swedish lakes
Source: Ambio. 2014 Nov 15;43(Suppl 1):19–29. doi: 10.1007/s13280-014-0559-y (PMC4235930; doi:10.1007/s13280-014-0559-y)
Supplement: Supplementary file 1 — Supplementary material 1 (PDF 72 kb) [file 13280_2014_559_MOESM1_ESM.pdf]

AMBIO
Electronic Supplementary Material
This supplementary material has not been peer reviewed

Title: **Challenges in assessing biological recovery from acidification in Swedish lakes**

Author: Kerstin Holmgren

Table S1. Descriptives of annual metrics for each of three acidified lakes, and results of trend test using Kendall's correlation. N is number of years in each time series. Significant trends ($P < 0.05$) are indicated in yellow when increasing and in blue when decreasing.

		Lake Brunnsjön							Lake Rotehogstjärnen							Lake Övre Skärsjön						
Parameter	Unit	N	Min	Max	Mean	SD	tau_b	P	N	Min	Max	Mean	SD	tau_b	P	N	Min	Max	Mean	SD	tau_b	P
Water temperature T(May-Sep)	°C	19	16.0	19.2	17.6	0.8	0.302	0.074	18	15.0	19.8	17.0	1.1	0.000	1.000	19	14.1	19.2	16.1	1.4	0.229	0.172
SO_4^{2-}	meq L^{-1}	19	0.121	0.329	0.198	0.054	-0.637	<0.001	19	0.042	0.168	0.089	0.042	-0.836	<0.001	19	0.064	0.146	0.099	0.028	-0,974	<0.001
Ca^{2+}	meq L^{-1}	19	0.159	0.248	0.190	0.023	-0.510	<0.001	19	0.054	0.111	0.075	0.017	-0.637	<0.001	19	0.056	0.092	0.073	0.012	-0,762	<0.001
Alkalinity/acidity (mean)	meq L^{-1}	19	-0.012	0.014	-0.000	0.007	0.422	0.012	19	-0.012	0.010	-0.001	0.006	-0.123	0.463	19	-0.003	0.014	0.005	0.004	0.446	0.008
Alkalinity/acidity (max)	meq L^{-1}	19	-0.066	0,000	-0.025	0.018	0.201	0.233	19	-0.043	-0.006	-0.024	0.010	-0.060	0.725	19	-0.012	0.010	-0.001	0.005	0.410	0.019
pH (mean)		19	5.25	5.70	5.50	0.11	0.387	0.021	19	5.21	5.74	5.46	0.13	-0.106	0.529	19	5.44	5.92	5.69	0.12	0.544	0.001
pH (min)		19	4.58	5.47	5.04	0.25	0.285	0.092	19	4.64	5.19	4.89	0.16	0.181	0.278	19	5.19	5.60	5.43	0.12	0.534	0.002
Conductivity (25 °C)	mS m^{-1}	19	5.27	7.96	6.03	0.70	-0.567	0.001	19	3.49	6.49	4.65	0.84	-0.474	0.005	19	2.19	3.40	2.65	0.44	-0.836	<0.001
Total N	µg L^{-1}	19	505	889	692	108	-0.053	0.753	19	358	556	435	56	-0.170	0.310	19	290	425	371	37	-0.193	0.248
Total P	µg L^{-1}	19	7.5	18.2	12.3	2.8	-0.164	0.327	19	6.5	15.1	12.9	2.2	-0.437	0.009	19	3.2	8.6	6.4	1.5	-0.448	0.008
AbsF(420 nm, 5 cm)		19	0.299	0.567	0.442	0.079	0.170	0.310	19	0.189	0.325	0.256	0.040	0.345	0.039	19	0.081	0.209	0.153	0.036	0.439	0.009
TOC	mg L^{-1}	19	15.2	26.9	20.4	2.8	0.263	0.115	19	9.7	17.0	12.6	1.8	0.439	0.009	19	5.9	10.8	8.1	1.3	0.532	0.001
Al_i (mean)	µg L^{-1}	16	26	90	55	17	-0.050	0.787	15	24	61	39	11	-0.048	0.805	16	16	54	27	10	-0.083	0,652
Al_i (max)	µg L^{-1}	16	57	279	100	55	-0.150	0.418	15	44	92	62	12	-0.162	0.400	16	18	121	47	27	0.109	0.558
Fish species richness	numbers	19	2	5	4.1	1.0	0.062	0.737	19	2	3	2.8	0.4	0.353	0.074	19	1	2	1.5	0.5	0.048	0.806
BPUE perch	g $gillnet^{-1}$	19	64	455	200	102	0.134	0.421	19	269	1118	628	206	-0.135	0.421	19	353	798	508	118	-0.368	0.028
BPUE roach	g $gillnet^{-1}$	19	20	250	93	55	-0.082	0.624	19	138	693	395	137	-0.462	0.006	19	0	5.2	0.3	1.2	0.216	0.273
MeanW perch	g	19	20	74	42	16	-0.287	0.086	19	21	54	38	8.4	-0.216	0.196	19	28	43	36	5	-0.141	0.401
MeanW roach	g	19	19	61	38	12	0.193	0.248	19	21	85	55	17	-0.415	0.013	1			172			
% age 1-3+ perch		19	14	85	59	24	0.333	0.046	19	35	74	54	12	0.112	0.506	18	21	67	49	13	-0.098	0.570
% age 1-3+ roach		18	0	53	19	17	0.079	0.649	18	3	93	50	27	-0.046	0.791	1			0			
L2+ perch	mm	17	94	126	107	9	0.090	0.620	19	100	136	112	11	-0.244	0.150	19	99	135	112	9	-0.359	0.033
bcL (0+) perch	mm	17	64	77	70	4	0.214	0.232	18	60	74	65	4	0.236	0.172	18	55	73	64	5	0.367	0.034
Chlorophyll a	µg L^{-1}	17	2.4	4.1	3.2	0.4	0.206	0.249	17	4.5	11.4	7.8	1.7	-0.265	0.138	17	1.6	4.7	2.2	0.7	-0.435	0.015
Phytoplankton taxa richness	numbers	19	30	54	39	5	0.000	1.000	19	22	58	44	8	0.203	0.232	19	31	53	39	7	0.316	0.065
Phytoplankton biovolume	$mm^3 m^{-3}$	19	93	781	274	148	0.158	0.345	19	189	2575	1283	593	-0.099	0.552	19	110	372	188	79	-0.006	0.972
% Chlorophyceae	%	19	2.5	42.3	17.7	9.1	-0.181	0.278	19	0.3	10.3	2.7	2.53	0.106	0.529	19	4.8	38.3	16.8	8.0	0.076	0.649
% Chrysophyceae	%	19	18.2	49.9	33.3	8.6	0.064	0.700	19	4.3	32.2	17.3	6.7	-0.018	0.916	19	19.1	45.8	31.9	7.1	-0.018	0.916
% Cryptophyceae	%	19	8.3	44.7	27.6	10.8	0.322	0.054	19	1.1	61.8	8.2	13.5	-0.263	0.115	19	3.7	22.9	10.9	6.1	0.333	0.046
% Dinophyceae	%	19	0.7	39.0	15.6	10.9	-0.216	0.196	19	0.5	8.6	3.1	2.4	-0.469	0.005	19	14.7	48.1	34.3	10.3	-0.018	0.916
% Raphidiophyceae	%	19	0.0	12.7	0.7	2.9	0.188	0.317	19	6.6	88.2	64.4	18.9	0.146	0.382	19	0.0	0.7	0.1	0.2	0.471	0.013
Zooplankton taxa richness	numbers	13	14	24	18	3	0.480	0.026	17	14	26	21	3	0.483	0.009	19	15	26	20	3	0.323	0.061
Zooplankton biovolume	$mm^3 m^{-3}$	13	514	2354	1048	490	-0.205	0.329	17	722	7648	2539	1872	0.250	0.161	19	214	1140	671	242	0.310	0.064
Rotatoria	$mm^3 m^{-3}$	13	7	191	48	57	0.385	0.067	17	168	6972	1866	1800	0.309	0.084	19	11	604	228	189	0.579	0.001
Cladocera	$mm^3 m^{-3}$	13	387	1904	647	436	-0.436	0.038	17	53	582	204	152	-0.015	0.934	19	135	564	313	142	0.088	0.600
Calanoid copepods	$mm^3 m^{-3}$	13	70	507	260	137	0.154	0.464	17	68	562	190	126	0.074	0.680	19	32	93	55	20	0.205	0.221
Cyklopoid copepods	$mm^3 m^{-3}$	13	30	163	93	41	-0.564	0.007	17	50	841	279	242	-0.250	0.161	19	32	246	76	50	-0.076	0.649
Benthic invertebrates taxa richness (3 habitats)	numbers	17	32	60	43	7	0.120	0.507	16	43	59	51	5	0.373	0.050	17	20	53	38	8	0.532	0.003
Benthic invertebrates (littoral)	n $sample^{-1}$	17	57	294	145	66	-0.044	0.805	16	153	1431	410	343	-0.033	0.857	17	22	277	151	80	0.162	0.365
Chironomidae (littoral)	n $sample^{-1}$	17	4	95	34	28	-0.324	0.070	16	27	797	186	206	0.217	0.242	17	2	73	20	19	0.294	0.099
Crustaceae (littoral)	n $sample^{-1}$	17	4	68	34	16	0.406	0.023	16	9	42	23	10	-0.293	0.115	17	4	121	44	32	0.480	0.007
Large insect larvae (littoral)	n $sample^{-1}$	17	22	135	50	31	-0.191	0.284	16	66	584	188	144	-0.250	0.177	17	8	152	51	36	0.132	0.458
Benthic invertebrates (sublittoral)	g m^{-2}	19	0.16	3.52	1.08	0.88	0.206	0.220	19	2.06	16.29	7.48	4.02	0.415	0.013	19	0.49	2.63	1.23	0.61	-0.211	0.208
Chironomidae (sublittoral)	g m^{-2}	19	0.05	2.45	0.44	0.60	0.271	0.107	19	1.31	15.77	5.65	3.94	0.251	0.132	19	0.10	1.84	0.59	0.51	-0.229	0.172
C. flavicans (sublittoral)	g m^{-2}	19	0,00	1.42	0.36	0.33	0.041	0.806	19	0.14	6.52	1.41	1.69	0.023	0.889	19	0.06	1.35	0.42	0.37	0.006	0.972
Benthic invertebrates (profundal)	g m^{-2}	19	0.69	8.02	4.30	2.12	0.380	0.023	19	6.85	39.38	17.15	7.53	0.070	0.674	19	0,00	6.02	0.71	1.37	-0.335	0.046
Chironomidae (profundal)	g m^{-2}	19	0.00	0.31	0.05	0.09	-0.100	0.576	19	0.6	11.83	3.26	3.20	-0.216	0.196	19	0,00	5.21	0.46	1.17	-0.237	0.160
C. flavicans (profundal)	g m^{-2}	19	0.69	8.00	4.25	2.12	0.380	0.023	19	3.42	36.97	13.88	7.72	0.240	0.151	19	0,00	1.31	0.18	0.35	-0.226	0.195
